# Exacerbated experimental arthritis in Wiskott–Aldrich syndrome protein deficiency: Modulatory role of regulatory B cells

**DOI:** 10.1002/eji.201344245

**Published:** 2014-07-15

**Authors:** Gerben Bouma, Natalie A Carter, Mike Recher, Dessislava Malinova, Marsilio Adriani, Luigi D Notarangelo, Siobhan O Burns, Claudia Mauri, Adrian J Thrasher

**Affiliations:** 1 Molecular Immunology Unit, UCL Institute of Child HealthLondon, UK; 2 Division of Medicine, UCL, Centre for Rheumatology ResearchLondon, UK; 3 Division of Immunology and The Manton Center for Orphan Disease Research, Children's Hospital, Harvard Medical SchoolBoston, MA, USA; 4 Department of Immunology, Great Ormond Street Hospital NHS TrustLondon, UK; 5 GlaxoSmithKline, ImmunoInflammation Therapy Area, Medicine Research CentreGunnels Wood Road, Stevenage, UK

**Keywords:** Arthritis, Immune regulation, Regulatory B cells, Regulatory T cell, Th17 cells, Wiskott–Aldrich syndrome protein

## Abstract

Patients deficient in the cytoskeletal regulator Wiskott–Aldrich syndrome protein (WASp) are predisposed to varied autoimmunity, suggesting it has an important controlling role in participating cells. IL-10-producing regulatory B (Breg) cells are emerging as important mediators of immunosuppressive activity. In experimental, antigen-induced arthritis WASp-deficient (WASp knockout [WAS KO]) mice developed exacerbated disease associated with decreased Breg cells and regulatory T (Treg) cells, but increased Th17 cells in knee-draining LNs. Arthritic WAS KO mice showed increased serum levels of B-cell-activating factor, while their B cells were unresponsive in terms of B-cell-activating factor induced survival and IL-10 production. Adoptive transfer of WT Breg cells ameliorated arthritis in WAS KO recipients and restored a normal balance of Treg and Th17 cells. Mice with B-cell-restricted WASp deficiency, however, did not develop exacerbated arthritis, despite exhibiting reduced Breg- and Treg-cell numbers during active disease, and Th17 cells were not increased over equivalent WT levels. These findings support a contributory role for defective Breg cells in the development of WAS-related autoimmunity, but demonstrate that functional competence in other regulatory populations can be compensatory. A properly regulated cytoskeleton is therefore important for normal Breg-cell activity and complementation of defects in this lineage is likely to have important therapeutic benefits.

Additional supporting information may be found in the online version of this article at the publisher's web-site

## Introduction

The Wiskott–Aldrich syndrome (WAS) is a rare X-linked primary immunodeficiency resulting from mutations in the gene encoding the Wiskott–Aldrich syndrome protein (WASp), a key regulator of the actin cytoskeleton in immune cells. Patients with WAS typically display recurrent infections, microthrombocytopenia, and eczema as well as a predisposition to develop autoimmunity and lymphoproliferative disease 1,2. Although 40–70% of patients with classical disease have been reported to develop autoimmunity 3,4, little is known about the underlying mechanisms and its development is likely to be multifactorial and related to dysfunction of different hematopoietic compartments 5. Several studies have revealed functional deficiency of natural T regulatory cells in both mouse models and human WAS patients 6–9. However, subsets of B cells have also emerged as important alternative regulators of autoimmunity (B regulatory cells, Breg). For example, mice are unable to resolve inflammation in experimental autoimmune encephalomyelitis (EAE) in the absence of IL-10 provided by autoreactive B cells 10,11. In addition, in models of arthritis and colitis, IL-10-producing B cells have been shown to possess disease modulating capability 12,13. Transfer of CD21^hi^CD23^hi^CD1d^hi^ transitional 2 marginal zone precursor (T2-MZP) B cells isolated either from naïve mice or from mice in the resolution phase of antigen-induced arthritis was sufficient to prevent the development of arthritis 14. T2-MZP Breg cells are profoundly depleted in MRL/lpr mice with established systemic lupus erythematosus 15. Similarly, Breg cells, identified as CD5^+^CD1d^hi^ B cells (B10 Breg cells), have been found to directly modulate EAE and murine systemic lupus erythematosus 16–18. WASp-deficient (WASp knockout [WAS KO]) mice have significantly reduced numbers of marginal zone (MZ) B cells and T2-MZP B cells reflecting marked disturbances of development and homeostasis of mature B-cell subsets 19–21. We therefore postulated that deficiency of Breg-cell function may contribute significantly to the autoimmune phenotype of WAS and used an experimental model of antigen-induced arthritis to investigate the contribution of Breg cells to WAS-related autoimmunity.

## Results

### Development of exacerbated experimental arthritis in WAS KO mice

**Figure 1:**
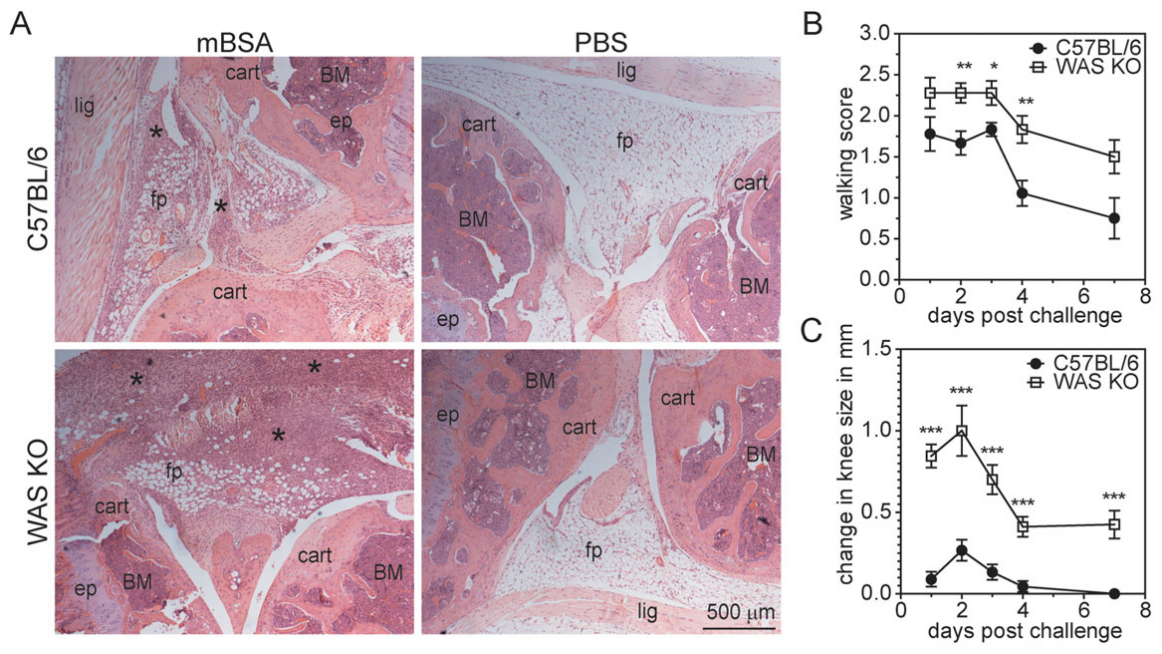
WAS KO mice develop exacerbated experimental arthritis. (A) H&E stainings of knee joints for cellular infiltrates in C57BL/6 and WAS KO mice, indicated by asterisks. Representative images are shown from day 7; cart: articular cartilage; ep: epiphyseal plate; fp: fat pad; lig: ligament. Scale bar 500 μm. (B, C) Severity of arthritis was assessed as (B) a clinical walking score and (C) knee swelling. Data are shown as mean ± SEM of nine animals per group up to day 4, and four animals per group at day 7; data are from a single experiment. *p < 0.05, **p < 0.01, ***p < 0.001, unpaired Student's *t*-test.

To study the development of autoimmune disease in WAS KO mice, we employed an experimental model of antigen-induced arthritis. Arthritis was induced by immunization with methylated BSA (mBSA), followed by intraarticular mBSA challenge. WAS KO mice developed exacerbated arthritis as shown by the accumulation of mononuclear cells in the knee joint compared with those in C57BL/6 animals (Fig.1A). This was accompanied by clinically more severe disease as assessed by a standardized walking score (Fig.1B) and measurement of knee joint swelling (Fig.1C). There was a highly significant positive correlation between the walking score and the amount of swelling observed in the mBSA-injected knee (Supporting Information Fig. 1A).

### Decreased numbers of Breg and Treg cells and increased Th17 cells in arthritic WAS KO mice

**Figure 2:**
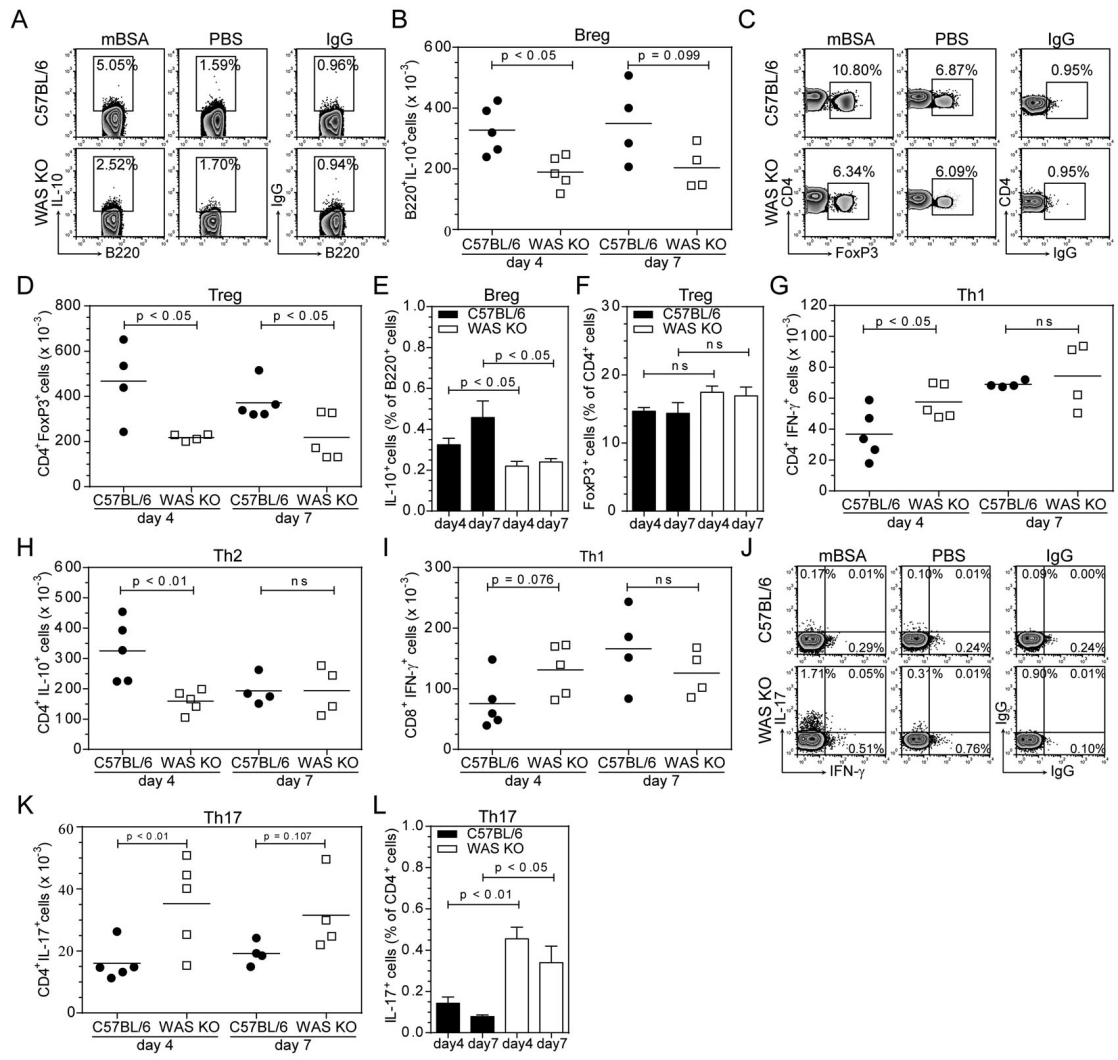
Decreased Breg cells and Treg cells and increased Th17 cells in experimental arthritis in WAS KO mice. (A) Breg cells were identified as B220^+^ cells expressing IL-10 by flow cytometry. IgG controls are shown in rightmost panels; (B) the data are presented as absolute cell numbers. (C) FoxP3 expression identified CD4^+^ Treg cells, IgG controls shown in rightmost panels; (D) the data are presented as absolute cell numbers. (E) Splenic Breg cells and (F) splenic Treg cells were analyzed by flow cytometry. (G) Quantification of IFN-γ-expressing CD4^+^ T cells, (H) IL-10-expressing CD4^+^ T cells, and (I) IFN-γ-expressing CD8^+^ T cells by flow cytometry. (J) Differential expression of IL-17 and IFN-γ, based on IgG controls (rightmost panel), of CD4^+^ lymphocytes was used to analyze and (K, L) quantify the presence of Th17 cells in (K) LNs or (L) spleen. Dot plots represent day 4 data and statistical significance was determined by Student's *t*-test. (B, D, G–I, K) Each symbol represents an individual mouse and bars represent means, from a single performed experiment. (E–F, L) Data are shown as mean ± SEM of *n* = 5 at day 4 and *n* = 4 at day 7 from a single experiment.

Next, we analyzed immune cell populations in the draining LNs of both the control and mBSA-injected knee joints. We observed a significant reduction in the number of both B220^+^IL-10^+^ cells (Fig.2A and B) and CD4^+^FoxP3^+^ Treg cells (Fig.2C and D) in WAS KO mice compared with those in C57BL/6 mice. The percentage of IL-10-producing B cells was also significantly reduced in spleens of arthritic WAS KO mice, whereas Treg-cell percentages were normal (Fig.2E and F). This is consistent with previous reports showing a variable defect of Treg-cell number with the most pronounced reduction in local LNs 6–9. There was an inverse correlation between the number of B220^+^IL-10^+^ B cells and Treg cells in the mBSA-knee-draining LNs and the severity of disease, both as an objective walking score and as the level of swelling observed in the mBSA-treated knee (Supporting Information Fig. 1B–E). Consistent with dampened Th1 response in IL-10^+^ B-cell sufficient arthritic WT C57BL/6 mice 14, we observed a trend for increased Th1 IFN-γ production (Fig.2G) and significantly reduced Th2 IL-10 production by CD4^+^ T cells from WAS KO mice (Fig.2H). No significant differences were observed for IFN-γ production by CD8^+^ T cells (Fig.2I).

As an important role in the development of autoimmune disease has been identified for IL-17-producing T (Th17) cells, we analyzed Th17 cells and found a significant increase of Th17 cells in LNs of mBSA-treated knees of WAS KO mice (Fig.2J and K). In the spleen, Th17 cells were also found in greater numbers (Fig.2L) and the number of Th17 cells correlated positively with the severity of disease (Supporting Information Fig. 1F–G).

### Reduced Breg cells under noninflammatory conditions

**Figure 3:**
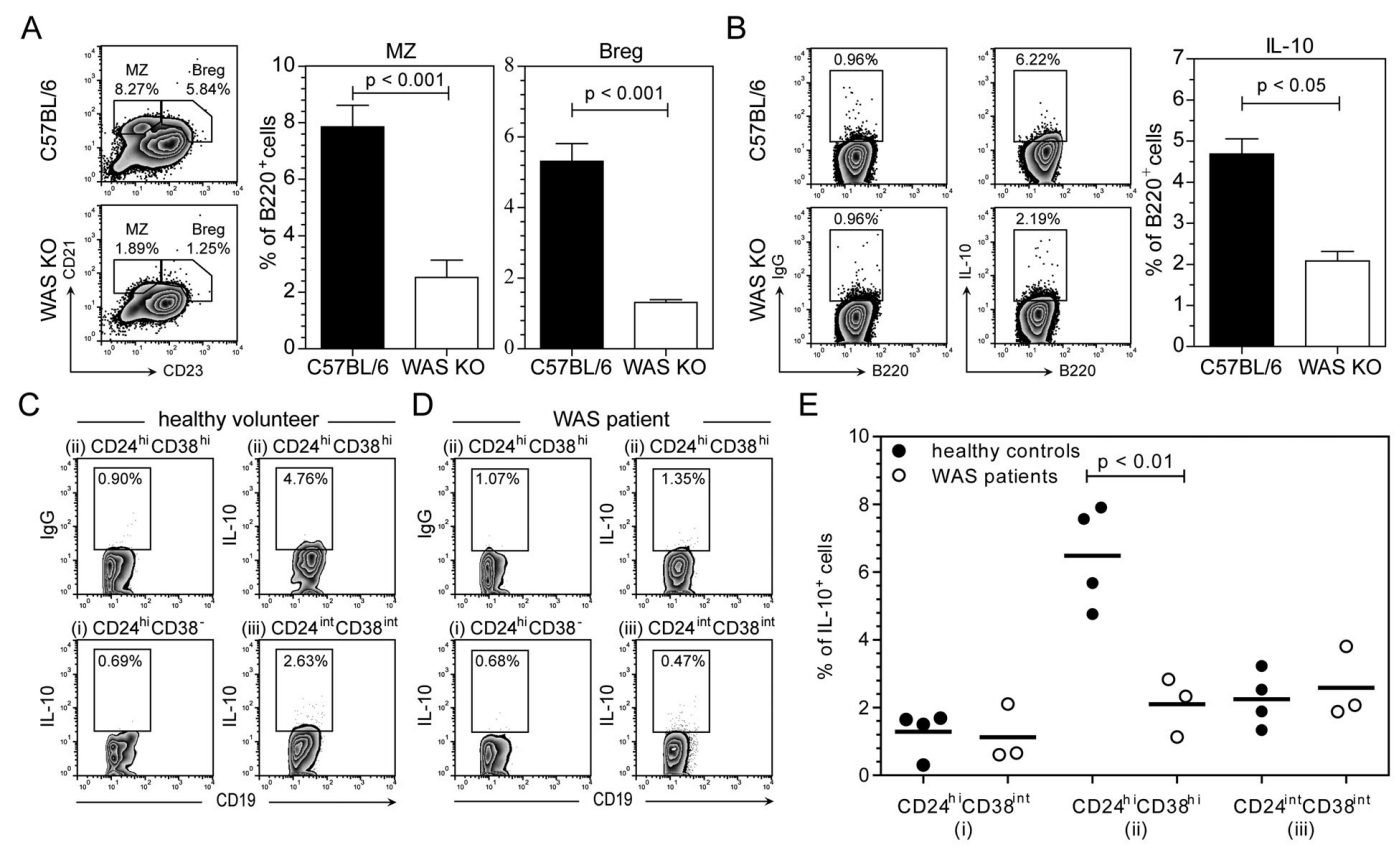
Fewer Breg cells under naive, noninflammatory conditions in spleens of WAS KO mice and blood of WAS patients. (A) Expression of CD21 and CD23 on B220^+^ B cells identified marginal zone (MZ) B and T2-MZP Breg cells (Breg, left). (B) Flow cytometric analysis of IL-10-producing B220^+^ B cells (IgG control left, IL-10 right). (C, D) Human B-cell subsets were gated as (i) CD24^hi^CD38^−^, (ii) CD24^hi^CD38^hi^, and (iii) CD24^int^CD38^int^ cells and their IL-10 expression was analyzed in (C) healthy volunteers and (D) WAS patients. Top right panel shows IgG control. (E) Quantification of IL-10-expressing B cells as shown in (C–D). (A, B) Data are shown as representative plots and mean ± SEM of *n* = 6 pooled from three experiments. (C–E) Data are shown as representative plots and with each symbol representing an individual healthy volunteer (*n* = 4) or WAS patient (*n* = 3), pooled from two experiments; bar represents mean. Statistical significance determined by unpaired Student's *t*-test.

IL-10-producing Breg cells have been found to be able to modulate autoimmune disease by affecting the frequency and equilibrium of both Treg cells and Th17 cells 22. Chimeric mice specifically lacking IL-10-producing B cells developed augmented arthritis, which was accompanied by a decrease of Treg cells and an increase of Th1 and Th17 cells 22. These findings therefore mirror our observations in WAS KO mice and suggest a numerical or functional deficiency. As in previous studies 19–21, we also observed a significant reduction in the number of B220^+^CD21^+^CD23^−^ MZ and also B220^+^CD21^+^CD23^+^ T2-MZP B cells (Fig.3A), with the latter containing Breg cells as identified by their IL-10 production (Fig.3B). As plasmacytoid DCs are also known to express B220 in mice, we also used CD19 to identify B cells and observed similar results (data not shown). In addition, when we identified Breg cells as CD19^+^CD5^+^CD1d^hi^ (B10 Breg) cells these were also reduced in number (Supporting Information Fig. 2A–B). These findings indicate that under noninflammatory conditions, B cells that have been identified to have regulatory activity are deficient in WAS KO mice.

In humans, a circulating CD19^+^CD24^hi^CD38^hi^ B-cell subset has been recently described that possesses regulatory function partially via provision of IL-10 23. Enumeration of this subset in younger children is complicated by the fact that these markers are shared with CD19^+^CD27^−^CD10^+^CD38^hi^ recent BM emigrant B cells, the frequency of which is higher at an early age (Supporting Information Fig. 3) 24. These markers alone therefore do not reliably identify a Breg population. We therefore stimulated PBMCs for 48 h with CpG and analyzed IL-10 production, which closely defines the population of B cells with regulatory activity. As before, we observed that the CD24^hi^CD38^hi^ B cells produced the most IL-10 (Fig.3C) 23. WAS patients (characterized by absent WASp expression, see Supporting Information Table 1) exhibited a significant reduction of IL-10-producing CD24^hi^CD38^hi^ B cells (Fig.3C–E), suggesting that there is a similar deficiency of B cells with regulatory activity to that observed in mice.

### Increased B-cell-activating factor levels, but unresponsiveness to BAFF stimulation in WAS KO mice

**Figure 4:**
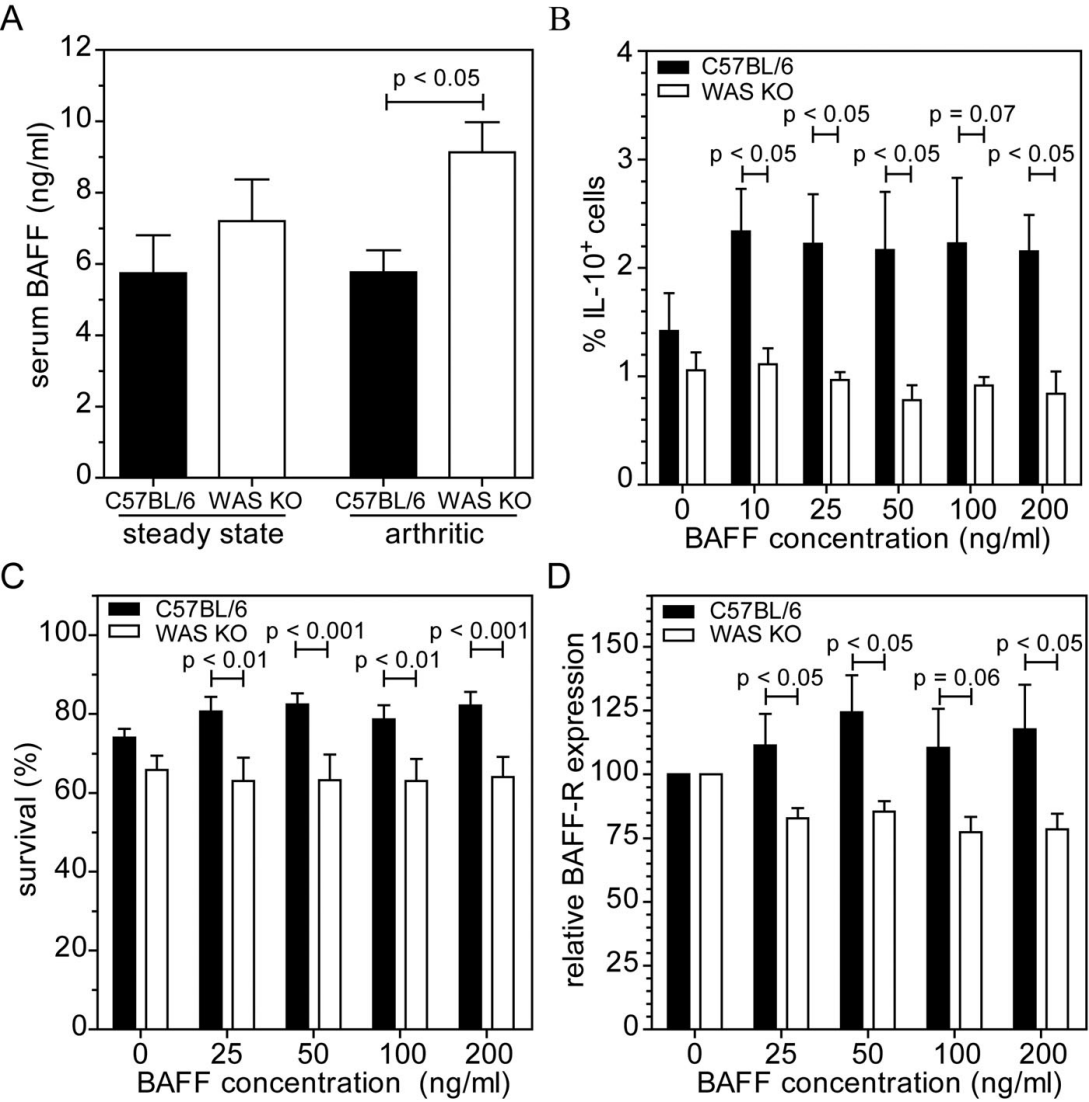
Increased serum BAFF levels in WAS KO mice, but unresponsiveness of WAS KO B cells to BAFF stimulation. (A) Serum levels of BAFF in healthy and arthritic C57BL/6 and WAS KO mice were measured by ELISA. (B) IL-10 expression in response to BAFF concentration in C57BL/6 and WAS KO mice was measured by flow cytometric analysis. (C) B-cell survival and (D) BAFF-R expression relative to 0 ng/mL BAFF was determined by flow cytometry. (A) Data are shown as mean + SEM of *n* = 8 C57BL/6 and *n* = 6 WAS KO mice for steady state and *n* = 3 for arthritic mice. (B–D) Data are shown as mean + SEM of *n* = 4, (C) *n* = 5 and (D) *n* = 6 mice pooled from three experiments. Statistical significance determined by unpaired Student's *t*-test.

B-cell-activating factor (BAFF) is an important signal promoting survival and differentiation of peripheral B cells. Mice transgenically overexpressing BAFF show a marked increase in transitional T2 and MZ B-cell numbers, while BAFF^−/−^ mice have strongly reduced numbers of transitional T2 and MZ B cells as well as follicular B cells 25,26. Furthermore, treatment of B cells with BAFF was shown to induce IL-10 production and a regulatory phenotype 27. As WAS KO mice have reduced MZ B and Breg cells, we set out to analyze whether defective BAFF production or function is involved. When we analyzed serum levels of BAFF, we however, found that BAFF levels in WAS KO mice were increased. This was most pronounced and significant in arthritic mice, while under noninflammatory conditions there was a trend for increased serum BAFF (Fig.4A). These findings were unexpected, as we would have expected increased MZ B and Breg cells in the presence of elevated BAFF levels 25,27. We therefore investigated whether WAS KO B cells are responsive to BAFF stimulation. When we stimulated B cells with BAFF, we observed that in contrast to WT B cells, WAS KO B cells do not produce IL-10, show reduced survival and fail to upregulate BAFF receptor expression (Fig.4B–D). These findings suggest that WAS KO B cells fail to effectively respond to BAFF stimulation and could indicate that the increased serum levels of BAFF reflect an uncoupling of a feedback mechanism regulating BAFF-mediated B-cell maturation.

### Adoptive transfer of Breg cells ameliorated disease

**Figure 5:**
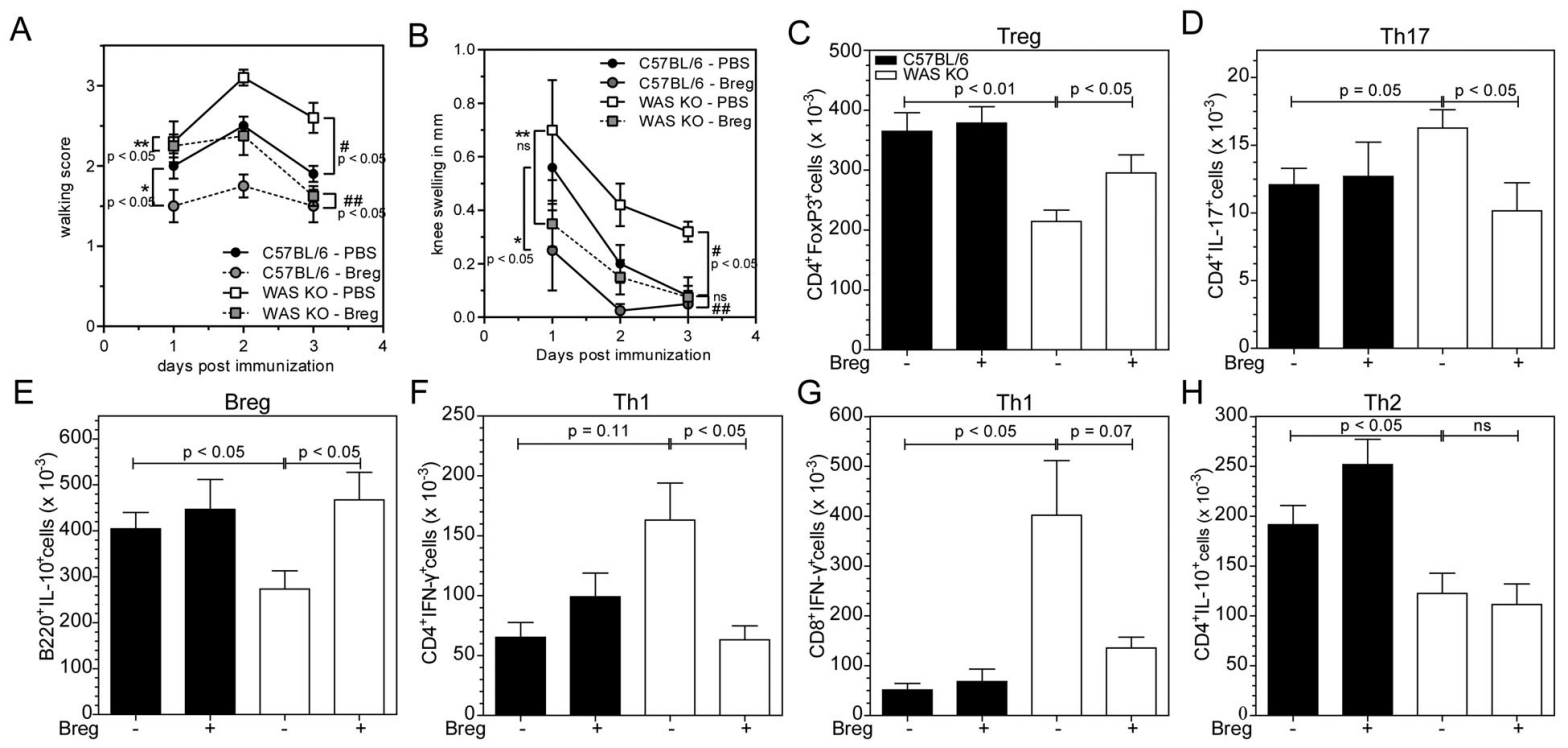
Transfer of T2 Breg cells ameliorates the development of arthritis in WAS KO mice. (A, B) Severity of experimental arthritis was assessed after Breg-cell transfer as (A) clinical walking score and (B) knee swelling. Data are shown as mean ± SEM from one experiment with control (PBS injected) *n* = 5, T2 Breg *n* = 4, #C57BL/6 PBS versus WAS KO PBS, ##C57BL/6 Breg versus WAS KO Breg, *C57BL/6 PBS versus C57BL/6 Breg, **WAS KO PBS versus WAS KO Breg (one-way ANOVA of AUC, *p* < 0.05). (C–H) The indicated cell populations in the draining LNs of mBSA-injected knees were analyzed by marker staining and flow cytometry. (C) Treg cells, (D) Th17 cells, (E) Breg cells, (F) IFN-γ-expressing CD4^+^ T cells and (G) CD8^+^ T cells and (H) IL-10-producing CD4^+^ T cells were quantified and shown as mean ± SEM of *n* = 5 (PBS transfer), and *n* = 4 (T2 Breg-cell transfer) from a single experiment performed. Statistical significance determined by Student's *t*-test.

To investigate whether the reduction in Breg cells contribute directly to altered Treg- and Th17-cell equilibrium and exacerbated arthritis in WAS KO mice, we isolated B220^+^CD21^+^CD23^+^ T2-MZP B cells from C57BL/6 mice with arthritis and adoptively transferred these into WAS KO mice prior to arthritis induction. Due to the low number of T2-MZP B cells present in WAS KO mice, we were only able to isolate and sort sufficient number of cells from control C57BL/6 mice for adoptive transfer experiments. Transfer of Breg cells into WAS KO mice ameliorated disease development significantly as shown by the reduced knee swelling and improved ability of the animals to walk (Fig.5A and B). Transfer of Breg cells into C57BL/6 animals also showed a modest but significant amelioration of disease severity (Fig.5A and B), as has been reported previously 14,22. After transfer of Breg cells, we observed an increase in the absolute number of Treg cells (Fig.5C) and a reduction in the number of Th17 cells in draining LNs of WAS KO mice (Fig.5D). The number of Breg cells also increased (Fig.5E), although our analysis did not allow us to distinguish whether this increase reflected the transferred cells or whether endogenous Breg cells were also induced. Transfer of Breg cells into WAS KO mice appeared to inhibit the Th1 response, although no effect on Th2 cytokine response was observed (Fig.5F–H). Our findings suggest that transfer of WT Breg cells at least partially overcomes the intrinsic Breg-cell deficiency of WAS KO mice.

### B-cell-restricted WASp deficiency is not sufficient to cause severe disease

**Figure 6:**
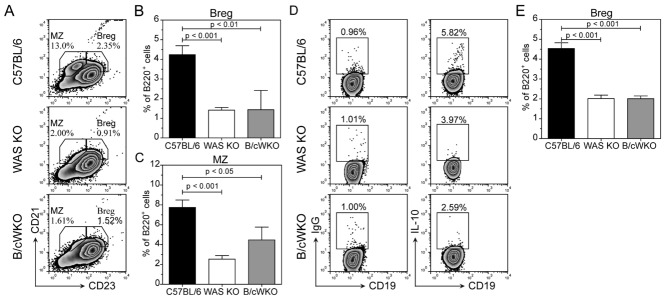
Reduced Breg cells in spleens of B/cWKO mice under naive, noninflammatory conditions. (A) Gating based on differential expression of CD21 and CD23 on B220^+^ B cells is shown for marginal zone B cells (MZ) and T2-MZP Breg cells (Breg). Representative dotplots are shown. (B, C) The number of (C) Breg cells and (C) MZ B cells in C57BL/6, WAS KO, and B/cWKO mice is shown as mean + SEM of *n* = 12 (C57BL/6), *n* = 11 (WAS KO), and *n* = 4 (B/cWKO) mice. (D) IL-10-expressing B cells isolated from spleens after stimulation with LPS for 24 h was assessed by flow cytometry. Top panels show IgG controls and bottom panels show gating for IL-10-producing CD19^+^ B cells. Representative dot plots are shown. (E)The percentage of IL-10-expressing B cells is shown as mean + SEM of *n* = 8 (C57BL/6), *n* = 8 (WAS KO), and *n* = 4 (B/cWKO) mice pooled from six experiments. Statistical significance determined by Student's *t*-test.

**Figure 7:**
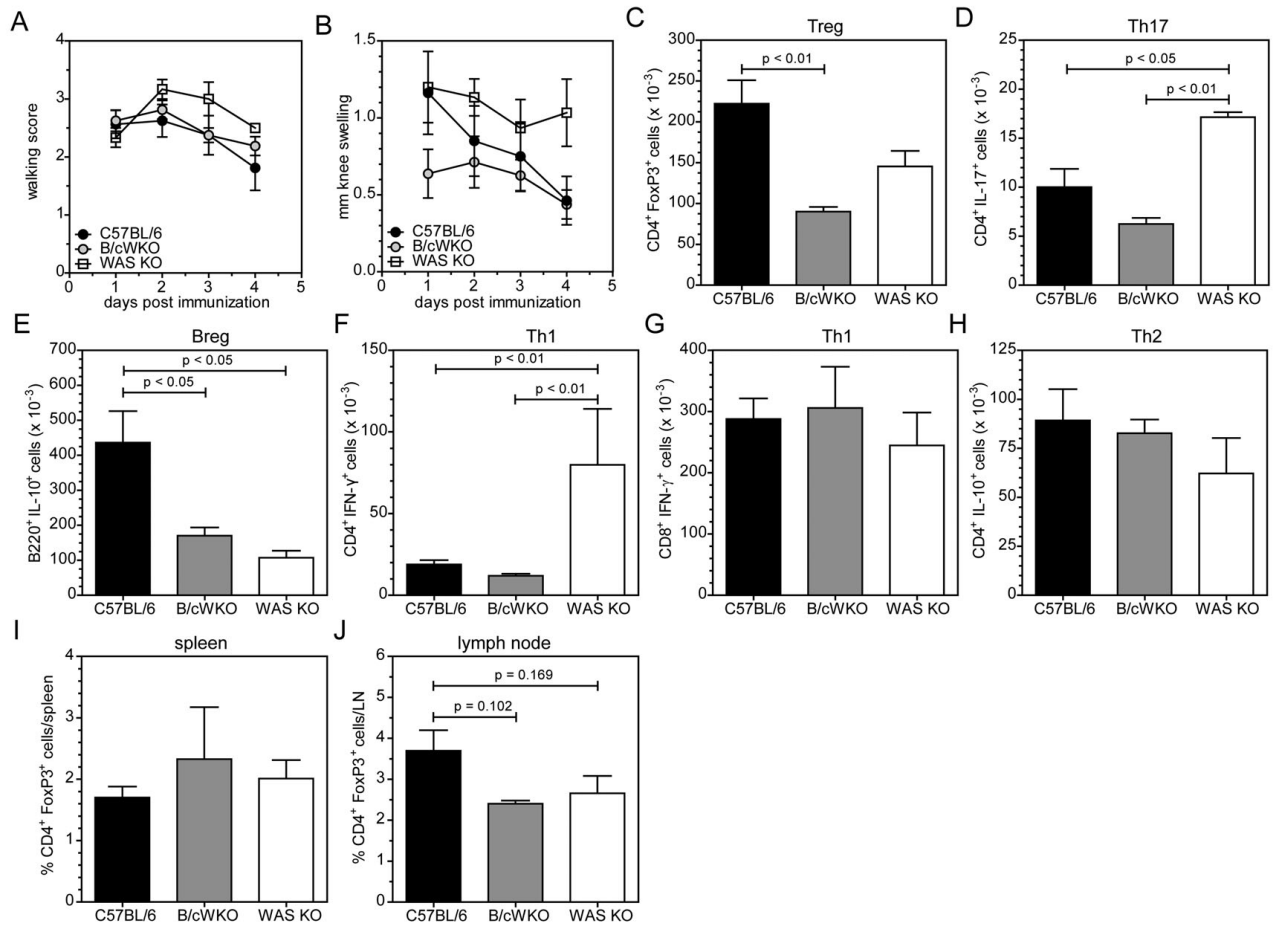
B/cWKO mice do not develop severe arthritis despite having reduced numbers of Breg cells. (A, B) Experimental arthritis development was assessed by (A) clinical walking score and (B) knee swelling. (C–H) The indicated cell populations in the draining LNs of mBSA-injected knees were analyzed by marker staining and flow cytometry. The numbers of (C) Treg cells, (D) Th17 cells, (E) Breg cells, (F) IFN-γ-expressing CD4^+^ T cells and (G) CD8^+^ T cells, and (H) IL-10-producing CD4^+^ T cells are shown as mean ± SEM of *n* = 8 (C57BL/6), *n* = 8 (B/cWKO), and *n* = 3 (WAS KO) mice pooled from two experiments. (I, J) The frequency of Treg cells were analyzed under noninflammatory conditions in (I) spleen and (J) LNs and shown as mean ± SEM of *n* = 5 (C57BL/6), *n* = 3 (B/cWKO), and *n* = 4 (WAS KO) mice pooled from two independent experiments. Statistical significance determined by Student's *t*-test.

Next, we set out to investigate whether the exacerbated arthritis is due to B-cell intrinsic defects. For this we used the novel model of B-cell selective WASp deficiency that was generated by crossing mice that have the *WAS* locus floxed by homologous recombination with *mb1*-Cre knock-in mice 28 These mice have a selective and efficient deletion of the *WAS* locus in the B-cell lineage effectively eliminating WASp expression (Supporting Information Fig. 4A) and, similar to WAS KO mice, exhibit reduced numbers of MZ B cells 28 and Breg cells (Fig.6A–E). Similar to mice with full WASp deficiency (Fig.4A), serum levels of BAFF tended to be increased under noninflammatory conditions in B/cWKO mice (8.06 ± 1.61 ng/mL, *n* = 5). After induction of arthritis, surprisingly, we found that B/cWKO mice develop disease of similar clinical severity as WT C57BL/6 mice (Fig.7A and B). Analysis of knee-draining LNs, however, showed that B/cWKO mice show reduced numbers of Breg and Treg cells, similar to WAS KO mice, but no increase in Th17 cells (Fig.7C–E). Serum BAFF levels in arthritic B/cWKO mice were also increased (9.80 ± 0.26 ng/mL, *n* = 3, *p* < 0.05 compared with those in C57BL/6, see Fig.4A), similar to WAS KO mice. In contrast, Th1 and Th2 responses in B/cWKO mice showed a phenotype that resembled WT C57BL/6 rather than WAS KO mice (Fig.7F–H). These results indicate that deficiency of WASp restricted to B cells was sufficient to generate defective Breg- and Treg-cell responses, but insufficient to cause severe Th17-mediated disease. Under noninflammatory conditions, Treg-cell numbers in B/cWKO mice were not significantly different from WT in spleen (Fig.7I) and LNs (Fig.7J) and suppressive function of B/cWKO Treg cells was found to be normal (Supporting Information Fig. 4B). This would suggest that modulation of Treg-cell numbers by B/cWKO Breg cells is specific to disease development, but may not be sufficient to modulate Treg-cell function.

## Discussion

Development of autoimmunity is a significant complication in the WAS with an incidence of 40–70% in patients reported 3,4. In addition, a large number of patients with mixed/split chimerism after hematopoietic stem cell transplantation develop autoimmunity, although the etiology and relationship, if any, to residual WASp deficiency is unclear 29,30. Here, we have studied the potential contribution of Breg cells to the control of immune homeostasis as MZ B cells are known to be significantly reduced in WAS KO mice and humans 19–21 and have recently been ascribed regulatory activity through IL-10 production 31. Under noninflammatory steady state conditions, WAS KO mice had fewer Breg cells in spleen, determined as IL-10-producing B cells and immunophenotypically as part of a CD21^+^CD23^+^ T2-MZP population. Furthermore, the numerical reduction in Breg cells was mimicked in the blood of WAS patients, where we observed a reduction in the number of IL-10-producing B cells. Here, we have used an experimental arthritis model to investigate autoimmunity in WAS KO mice. We observed that WAS KO mice developed exacerbated arthritis, which correlated with a decrease in Breg and Treg cells and increase in Th17 cells in draining LNs and spleen. Furthermore, there was a trend for Th1 cells to also be increased in WAS KO mice. Using adoptive transfer, we found that restoration of Breg-cell activity in the early phase of arthritis development led to an increase in the number of Treg cells and concomitant decrease in the number of Th17 cells, resulting in ameliorating of disease. This exactly mirrors our previous findings using chimeric mice specifically lacking IL-10-producing B cells that showed exacerbated autoimmune disease, reduced Treg cells, and increased Th1- and Th17-cell numbers 22. In this model, transfer of WT CD21^+^CD23^+^ T2-MZP Breg cells (but not CD21^+^CD23^−^ MZ or CD21^int^CD23^int^ follicular B-cell subsets) also increased Treg cells and reduced Th1- and Th17-cell frequencies resulting in inhibition of inflammation. These findings suggest a prominent role for IL-10-producing Breg cells in modulating disease severity and for control of autoimmune of autoinflammatory processes.

In contrast to WAS KO mice, mice with B-cell-specific WASp deficiency 28 surprisingly showed a milder disease phenotype similar to WT C57BL/6 mice, despite showing a reduction in the number of Breg and Treg cells in the LNs draining affected knees. However, contrary to WAS KO mice, we did not find increased numbers of Th17 cells in the knee-draining LNs of arthritic B/cWKO mice, suggesting that other regulatory compartments are compensating. For example, WAS KO mice have defective Treg-cell function 6–9, whereas in the B/cWKO model, Treg cells express WASp normally and have normal intrinsic functionality. Furthermore, under noninflammatory conditions, Treg-cell numbers in both B/cWKO and WAS KO mice were found to be normal in spleen and only slightly (not significantly) reduced in LNs. Our findings are therefore supportive of a role for Breg cells modulating the number of Treg cells during a state of active immune activity and are consistent with previous observations for such a role of Breg cells in other experimental arthritis models 14,22, and also in allergen-induced airway inflammation and EAE 32,33. The precise contribution of Treg cells in experimental arthritis in WASp deficiency remains to be investigated. Furthermore, both intrinsic T-cell differentiation 34,35 and extrinsic T-cell priming by antigen-presenting DCs 36,37 are known to be defective in WASp deficiency, which may contribute to the aberrant induction of Th17 cells. Th17 cells are known to play an important role for arthritis severity and progression 38 and our data supports a key role for these cells in the development of severe arthritis in WASp deficiency.

We observed increased serum levels of BAFF in both WAS KO and B/cWKO mice and increased serum BAFF expression was also recently reported in WAS patients 39. Overexpression of BAFF in mice results in a marked increase in transitional T2 and MZ B-cell numbers, while BAFF^−/−^ mice show reduced numbers of transitional T2 and MZ B cells 25,26. Increased BAFF levels in WAS KO mice were therefore unexpected. However, WAS KO B cells appeared to exhibit reduced responsiveness to BAFF stimulation, which would suggest that the increased serum levels of BAFF, in particular in arthritic mice could be part of a failing feedback mechanism attempting to increase the number of mature B-cell populations. Deletion of Cdc42, the main effector of WASp, has been described to result in significantly reduced mature B-cell populations and responsiveness to BAFF stimulation 40, favoring involvement of WASp in BAFF receptor signaling events, but this remains to be investigated.

Several studies have shown that WAS KO mice develop autoantibodies and their presence has been linked with the development of renal histopathology 7,41,42. Similarly, increased autoantibody production and autoimmunity have been demonstrated in two models of B-cell-selective WASp deficiency suggesting that there is a B-cell intrinsic role in WAS-related autoimmunity 28,43. How this influences other autoreactive cell populations is unclear. However, a subset of CD5^+^ B cells has been identified to express high levels of Fas ligand 44. These cells also expressed IL-10 and further studies identified these as Breg cells that played an important role in inducing apoptosis of antigen-specific T cells, by cell–cell contact as well as Fas ligand secretion 45. Moreover, CD5^+^ Fas ligand positive Breg cells were implicated in immune regulation in collagen-induced arthritis by promoting apoptosis in arthritis-associated T cells 46.

In summary, our data demonstrate that the cytoskeletal regulator WASp is required for acquisition of normal Breg-cell numbers and for normal function. This may have an important influence on the balance and recruitment of Treg and Th17 cells during inflammation. The mechanism by which Breg cells influence experimental arthritis is not fully clear, although local secretion of immunosuppressive cytokines such as IL-10, or control of T-cell apoptosis remains attractive possibilities. Interestingly, selective deficiency of WASp in Breg cells appears to be compensated by intact functionality in other regulatory lineages, suggesting that partial restoration of regulatory cell function in WAS may be sufficient to protect or alleviate autoimmunity. Overall, it seems likely that autoimmunity in WAS has a multicompartmental etiology and that the contribution from different cell lineages may depend on context and type of process involved.

## Materials and methods

### Mice

WAS KO and B/cWKO mice were bred in our own facilities and C57BL/6 were purchased from Harlan. Mice were used at 6–12 weeks of age and all experiments approved by and performed according to Home Office Animal Welfare Legislation (PPL 70/7329).

### Patients

Peripheral blood was obtained from WAS patients (Supporting Information Table 1) and controls following informed consent obtained in accordance with the Declaration of Helsinki and with ethical approval from the Great Ormond Street Hospital for Children and the Institute of Child Health Research Ethics. Human PBMCs were isolated using Ficoll-Hypaque (Amersham Pharmacia, Little Chalfont, UK) gradient centrifugation.

### Induction and assessment of arthritis

Mice were immunized s.c. with endotoxin-free mBSA (1 μg/mL; Sigma-Aldrich, Steinheim, Germany) in complete Freund's adjuvant and 7–10 days later challenged by intraarticular injection of 10 μL mBSA (20 μg/mL) in one knee and PBS in the other knee. The development of arthritis was assessed daily in a blinded manner. Clinical severity was determined by assessment of the ability of the animals to walk normally (walking score) and graded as follows: 0, normal; 1, limping; 2, severe limping causing the animal to hop; 3, refusal to put weight on leg; 4, refusing to walk at all. In addition, swelling of both knee joints was measured daily using calipers (knee swelling). Animals were sacrificed at days 4 and 7 for analysis of immune cell populations in draining LNs and spleen. Knee joints were removed postmortem, fixed in 10% formalin (Sigma-Aldrich), embedded in paraffin followed by sectioning and H&E staining.

### Flow cytometric analysis

Single-cell suspensions were prepared from spleen and inguinal LNs and cultured in RPMI with 10% FCS supplemented with 100 units/mL penicillin and 100 μg/mL (Invitrogen, Paisley, UK). For analysis of steady state Breg cells, splenocytes were stained with antibodies against CD21 (FITC), CD23 (PE), B220 (allophycocyanin). All antibodies were from BD Biosciences (Erembodegem, Belgium), unless stated otherwise. For analysis of IL-10-producing cells, splenocytes or CD19-selected B cells (CD19 selection beads, Miltenyi Biotec, Bergisch Gladbach, Germany) were stimulated with 100 ng/mL LPS (Sigma-Aldrich) for 24 h and PMA (50 ng/mL; Sigma-Aldrich), ionomycin (500 ng/mL; Sigma-Aldrich), and brefeldin A (5 μg/mL; Sigma-Aldrich) were added for the last 4–6 h. Cells were then stained with PerCP-conjugated anti-B220, followed by permeabilization (BD Fix/Perm) and intracellular stain with APC-conjugated anti-IL-10 or APC-conjugated isotype control. For analysis of BAFF stimulation, CD19-selected B cells were stimulated with the indicated concentrations of recombinant mouse BAFF (Enzo Life Sciences, Exeter, UK) for 72 h followed by analysis of cell viability using 7AAD staining, analysis of BAFF-R (eBioscience, San Diego, CA, USA) and IL-10 expression by flow cytometry. Cells from arthritic mice were cultured in vitro for 5 h with PMA, ionomycin, and brefeldin A and then stained with antibodies against B220 (PerCP), CD4 (FITC), CD8 (PerCP), and CD25 (PE) followed by permeabilization (BD Fix/Perm or FoxP3-specific permeabilization reagents from eBioscience) and intracellular staining for FoxP3 (allophycocyanin; eBioscience), IL-17 (PE; Miltenyi Biotec), IFN-γ (allophycocyanin), or appropriately conjugated isotype controls. For analysis of B10 Breg cells, splenocytes were stained with PE-Cy7 anti-CD19, PE anti-CD1d, and FITC anti-CD5. Human PBMC was stained with antibodies against CD19 (PerCP-Cy5.5), CD24 (FITC), and CD38 (PE) or stimulated for 48 h with 0.1 μM CpG (Invivogen, San Diego, CA, USA) and the last 4–6 h with PMA, ionomycin, and brefeldin A followed by staining with antibodies against CD19 (PerCP-Cy5.5), CD24 (FITC), CD38 (PE), permeabilization (BD Fix/Perm), and intracellular staining with anti-IL-10 (allophycocyanin). Cells were acquired with a Cyan cytometer (Dako Cytomation, Glostrup, Denmark) and analyzed with FlowJo software (Treestar, Ashland, OR, USA).

### Adoptive transfer

Splenocytes were isolated from 19 C57BL/6 mice that were preimmunized with mBSA and challenged intraarticularly with mBSA as described above, and depleted for CD43^+^ cells using microbeads and LD columns (Miltenyi Biotec). The resulting cells were stained with anti-CD21-FITC, anti-CD23-PE, anti-CD4-biotin, streptavidin-allophycocyanin, and CD19-PE-Cy7 (BD Biosciences) and sorted by a FACS Aria cell sorter (Beckman Coulter, Brea, CA, USA) for CD19^+^CD21^+^CD23^+^CD24^hi^ Breg cells that were subsequently injected (1.7 × 10^6^) into the tail veins of C57BL/6 or WAS KO recipients at the time of intraknee mBSA challenge. Development of arthritis was assessed daily by knee swelling measurement and clinical assessment and animals were sacrificed at day 3 for analysis of immune cell populations in the draining LNs.

### T-cell suppression

CD4^+^CD25^hi^ natural Treg cells and CD4^+^CD25^−^ target cells were purified from spleens of B/cWKO and control littermates by flow cytometry as previously reported 6. T-cell-depleted spleen cells (feeder cells) were obtained from control splenocytes by magnetic depletion of CD3^+^ cells (Miltenyi Biotec). Suppression assays were performed in triplicate using 25 000 CD4^+^CD25^−^ target cells from control littermate spleens, 250 000 irradiated (30 Gy) feeder cells and 25 000 (ratio 1:1) or 5000 (ratio 1:5) CD4^+^CD25^+^ natural T regulatory cells from B/cWKO or C57BL/6 mice in the presence of soluble anti-CD3 and anti-CD28 Abs (1 μg/mL each; BD Biosciences). After 4 days, cultures were pulsed with μCi [3H]thymidine (GE Healthcare, Buckinghamshire, UK) for 16 h before harvesting and measurement of the incorporated radioactivity by scintillation counting (Perkin Elmer, Buckinghamshire, UK).

### Statistical analysis

Data are expressed as mean ± SEM and analyzed using Student's *t*-test or one-way ANOVA of the area under the curve. All statistical tests were performed using Prims 5 (GraphPad). *p* Values < 0.05 were considered as statistically significant.

## Abbreviations

BAFF: B-cell-activating factor
Breg cell: regulatory B cell
EAE: experimental autoimmune encephalomyelitis
MZ: marginal zone
mBSA: methylated BSA
T2-MZP: transitional 2 marginal zone precursor
WAS: Wiskott–Aldrich syndrome
WASp: Wiskott–Aldrich syndrome protein
WAS KO: WASp knockout

## Supporting Information

As a service to our authors and readers, this journal provides supporting information supplied by the authors. Such materials are peer reviewed and may be re-organized for online delivery, but are not copy-edited or typeset. Technical support issues arising from supporting information (other than missing files) should be addressed to the authors.

**Figure S1**. Severity of arthritis correlates with cell populations (A) The severity of arthritis shows positive linear correlation of walking score and knee swelling, data of mBSA knee swelling of C57BL/6 and WAS KO combined, data obtained at days 0, 1, 2, 3, 4 (n = 18) and 7 (n = 8), p value is indicated (Pearsons r). Severity of arthritis assessed as knee swelling correlates negatively with the number of (B) Breg cells and (C) Treg cells, and positively with (D) Th17 T cells in the draining LN of mBSA-injected knees. Severity of arthritis assessed as clinical walking score correlates negatively with the number of (E) Breg cells and (F) Treg cells, and positively with (G) Th17 T cells in the draining LN of mBSA-injected knees. P values are indicated (Pearsons r), data of C57BL/6 (day 4 n = 5 and day 7 n = 4) and WAS KO (day 4 n = 5 and day 7 n = 4) combined. **Figure S2**. Reduced B10 Breg cells in WAS KO mice (A) Differential expression of CD5 and CD19 on splenocytes shows the gating strategy for analysis of splenic CD19+CD5+CD1dhi B10 Breg cells. (B) Quantification of data in (A). Representative plots and quantification are shown of n = 4. Data is shown as averages ± SEM and p values are indicated (unpaired Student's t-test). **Figure S3**. Quantification of human B-cell subsets For analysis of human cells, PBMC were isolated and stimulated for 48 hrs with 0.1 μM CpG. PMA/ionomycin was added for the last five hrs and cells were stained for the expression of CD19, CD24, CD38 and IL-10. B-cell subsets were gated as CD24hiCD38- (i), CD24hiCD38hi (ii) and CD24intCD38int (iii) cells and their frequency quantified. Each symbol denotes an individual patient or healthy volunteer. **Figure S4**. Selective deficiency of WASp, restricted to the B-cell lineage (A) Flow cytometry analysis of WASp expression confirms B-cell-restricted WASp deficiency. Representative plots are shown of n = 4. (B) T-cell suppressive function was analyzed by co-culturing Treg cells with CD4+CD25- target cells and proliferation assessed by 3H-thymidine incorporation. Data is shown as averages ± SD, n = 3 in one experiment. **Table S1**. Patient characteristics.
