# Supplementary material for: Exacerbated experimental arthritis in Wiskott–Aldrich syndrome protein deficiency: Modulatory role of regulatory B cells
Source: Eur J Immunol. 2014 Jul 15;44(9):2692–702. doi: 10.1002/eji.201344245 (PMC4209796; doi:10.1002/eji.201344245)
Supplement: Supplementary file 1 — Figure S1. Severity of arthritis correlates with cell populations (A) The severity of arthritis shows positive linear correlation of walking score and knee swelling, data of mBSA knee swelling of C57BL/6 and WAS KO combined, data obtained at days 0, 1, 2, 3, 4 (n = 18) and 7 (n = 8), p value is indicated (Pearsons r). Severity of arthritis assessed as knee swelling correlates negatively with the number of (B) Breg cells and (C) Treg cells, and positively with (D) Th17 T cells in the draining LN of mBSA-injected knees. Severity of arthritis assessed as clinical walking score correlates negatively with the number of (E) Breg cells and (F) Treg cells, and positively with (G) Th17 T cells in the draining LN of mBSA-injected knees. P values are indicated (Pearsons r), data of C57BL/6 (day 4 n = 5 and day 7 n = 4) and WAS KO (day 4 n = 5 and day 7 n = 4) combined. Figure S2. Reduced B10 Breg cells in WAS KO mice (A) Differential expression of CD5 and CD19 on splenocytes shows the gating strategy for analysis of splenic CD19+CD5+CD1dhi B10 Breg cells. (B) Quantification of data in (A). Representative plots and quantification are shown of n = 4. Data is shown as averages ± SEM and p values are indicated (unpaired Student's t-test). Figure S3. Quantification of human B-cell subsets For analysis of human cells, PBMC were isolated and stimulated for 48 hrs with 0.1 μM CpG. PMA/ionomycin was added for the last five hrs and cells were stained for the expression of CD19, CD24, CD38 and IL-10. B-cell subsets were gated as CD24hiCD38- (i), CD24hiCD38hi (ii) and CD24intCD38int (iii) cells and their frequency quantified. Each symbol denotes an individual patient or healthy volunteer. Figure S4. Selective deficiency of WASp, restricted to the B-cell lineage (A) Flow cytometry analysis of WASp expression confirms B-cell-restricted WASp deficiency. Representative plots are shown of n = 4. (B) T-cell suppressive function was analyzed by co-culturing Treg cells with CD4+CD25- target cells [file eji0044-2692-sd1.pdf]

European Journal of Immunology

Supporting Information

for

DOI 10.1002/eji.201344245

Gerben Bouma, Natalie A. Carter, Mike Recher, Dessislava Malinova, Marsilio Adriani, Luigi D. Notarangelo, Siobhan O. Burns, Claudia Mauri and Adrian J. Thrasher

Exacerbated experimental arthritis in Wiskott–Aldrich syndrome protein deficiency: Modulatory role of regulatory B cells

© 2014 The Authors. *European Journal of Immunology* published by WILEY-VCH Verlag GmbH & Co. KGaA, Weinheim

Supporting Information

Exacerbated experimental arthritis in Wiskott-Aldrich syndrome protein deficiency: investigation of the modulatory role of regulatory B cells

Gerben Bouma[1*], Natalie A. Carter[2], Mike Recher[3], Dessislava Malinova[1], Marsilio Adriani[1], Luigi D. Notarangelo[3], Siobhan O. Burns[1,4], Claudia Mauri[2] and Adrian J. Thrasher[1,4]

Table 1. Patient characteristics

Patient	Mutation[1]	WASp expression	Score[2]	Autoimmunity	Follow up
1	p.Gly423CysfsX73	absent	3-4	no	BMT, thriving well
2	p.Gln91X	absent	3-4	no	BMT, thriving well
3	R34X	absent	3-4	no	GT, thriving well

[1] amino acid mutations, [2] based on Zhu et al [1], BMT, bone marrow transplant, GT, gene therapy

References

1 **Zhu, Q., Zhang, M., Blaese, R. M., Derry, J. M., Junker, A., Francke, U., Chen, S. H. and Ochs, H. D.,** The Wiskott-Aldrich syndrome and X-linked congenital thrombocytopenia are caused by mutations of the same gene. *Blood* 1995. **86**: 3797-3804.

Fig. 1

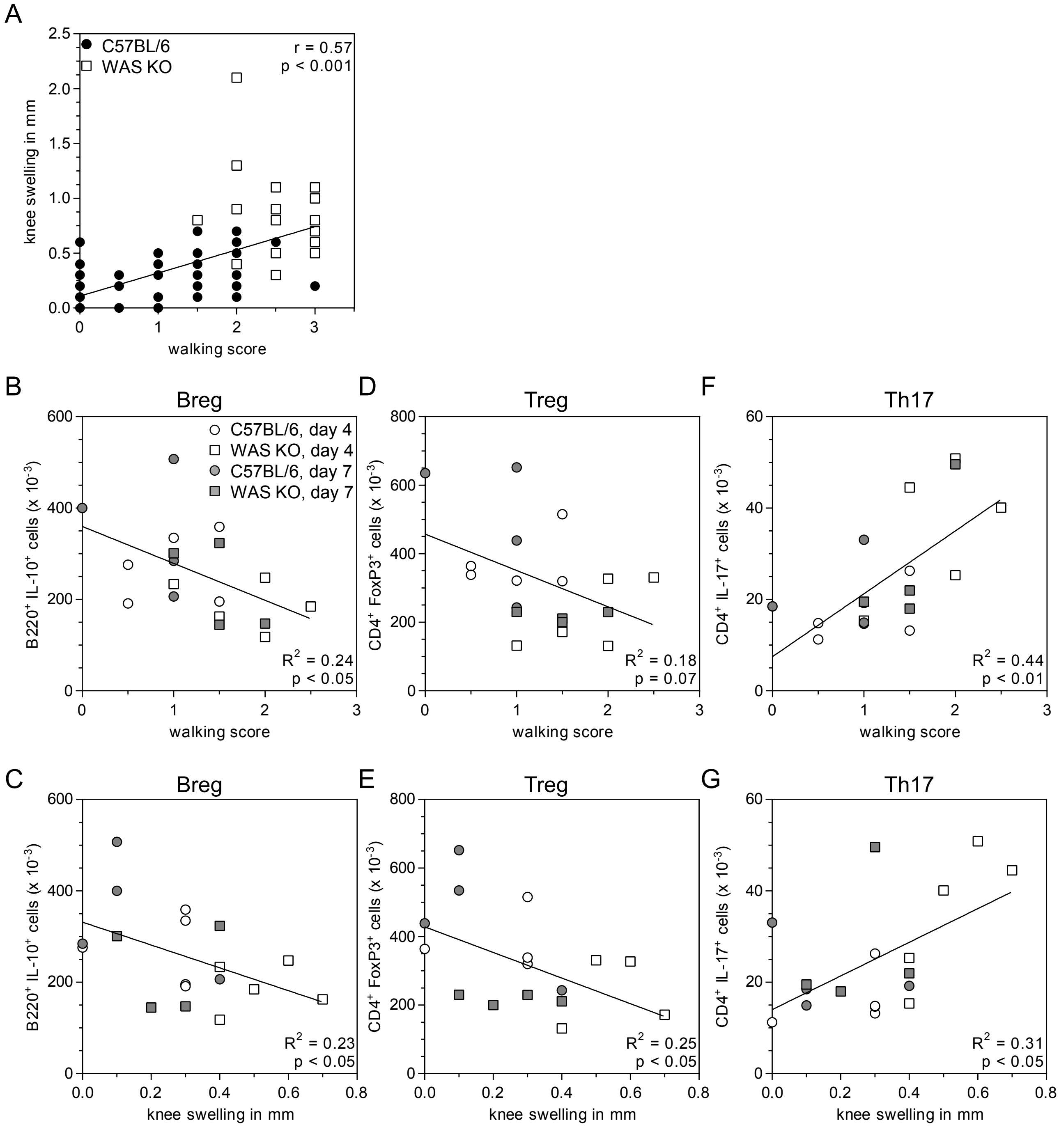

Fig. 1. Severity of arthritis correlates with cell populations
(A) The severity of arthritis shows positive linear correlation of walking score and knee swelling, data of mBSA knee swelling of C57BL/6 and WAS KO combined, data obtained at day 0, 1, 2, 3, 4 (n=18) and 7 (n=8), p value is indicated (Pearsons r). Severity of arthritis assessed as knee swelling correlates negatively with the number of (B) Breg cells and (C) Treg cells, and positively with (D) Th17 T cells in the draining lymph node of mBSA injected knees. Severity of arthritis assessed as clinical walking score correlates negatively with the number of (E) Breg cells and (F) Treg cells, and positively with (G) Th17 T cells in the draining lymph node of mBSA injected knees. P values are indicated (Pearsons r), data of C57BL/6 (day 4 n=5 and day 7 n=4) and WAS KO (day 4 n=5 and day 7 n=4) combined.

Fig. 2

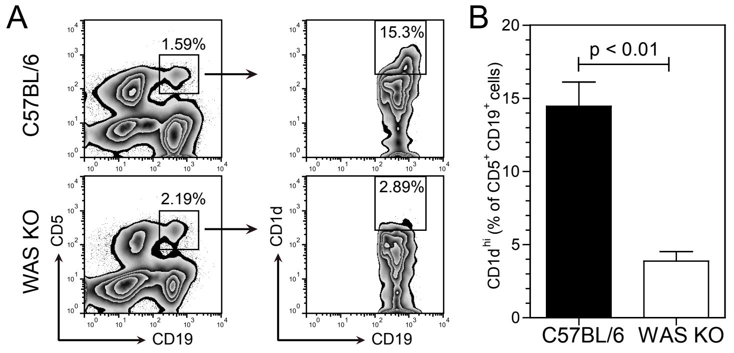

Fig. 2. Reduced B10 Breg cells in WAS KO mice (A) Differential expression of CD5 and CD19 on splenocytes shows the gating strategy for analysis of splenic CD19+CD5+CD1dhi B10 Breg cells. (B) Quantification of data in (A). Representative plots and quantification are shown of n=4. Data is shown as averages ± SEM and p values are indicated (unpaired Student's t-test).

Fig. 3

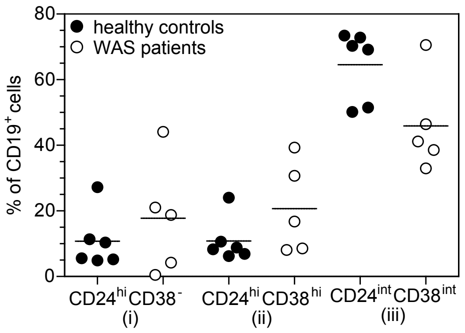

Fig. 3. Quantification of human B cell subsets
For analysis of human cells, PBMC were isolated and stimulated for 48 hrs with 0.1 μM CpG. PMA/ionomycin was added for the last five hrs and cells were stained for the expression of CD19, CD24, CD38 and IL-10. B cell subsets were gated as CD24hiCD38- (i), CD24hiCD38hi (ii) and CD24intCD38int (iii) cells and their frequency quantified. Each symbol denotes an individual patient or healthy volunteer.

Fig. 4

A

B

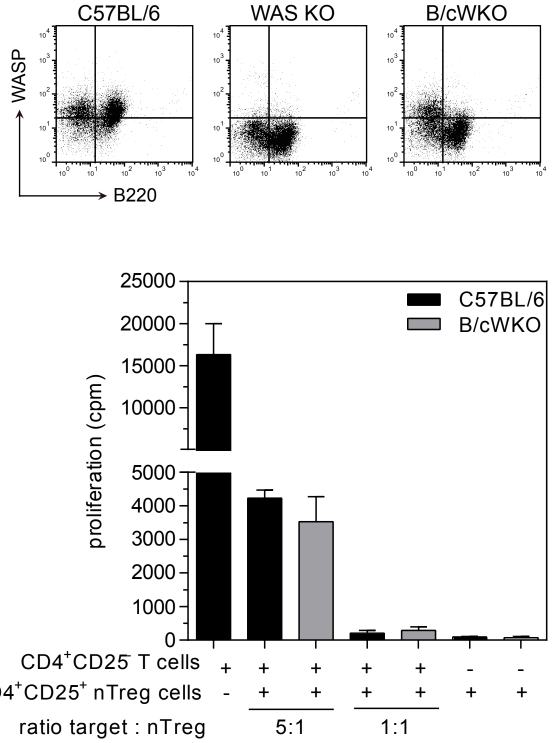

Fig. 4. Selective deficiency of WASp, restricted to the B cell lineage
(A) Flow cytometry analysis of WASp expression confirms B cell restricted WASp deficiency. Representative plots are shown of n=4. (B) T cell suppressive function was analysed by co-culturing Treg cells with CD4+CD25- target cells and proliferation assessed by 3H-thymidine incorporation. Data is shown as averages ± SD, n=3 in one experiment.
